# Retroperitoneal Renal Surgery with the Hugo™ RAS System: Feasibility, Setting and Perioperative Outcomes of the First Dedicated Series

**DOI:** 10.3390/jcm15155975

**Published:** 2026-07-31

**Authors:** Andrea Iannuzzi, Benito Fabio Mirto, Fabio Machiella, Francesco Smarrazzo, Giuseppe Quarto, Giuseppe Severini, Dario Cerasi, Alberto Ragusa, Francesco Tedesco, Francesco Prata, Felice Crocetto, Ciro Imbimbo, Rocco Papalia, Donato Dente

**Affiliations:** 1 Research Unit of Urology, Department of Medicine and Surgery, Università Campus Bio-Medico di Roma, 00128 Rome, Italy; 2 Department of Urology, Pineta Grande Hospital, 81030 Castel Volturno, Italy; fabio.mirto@pinetagrande.it (B.F.M.); fabio.machiella@pinetagrande.it (F.M.); francesco.smarrazzo@pinetagrande.it (F.S.); giuseppe.quarto@pinetagrande.it (G.Q.); giuseppe.severini@pinetagrande.it (G.S.); dario.cerasi@pinetagrande.it (D.C.); donato.dente@pinetagrande.it (D.D.); 3 Department of Urology, Fondazione Policlinico Universitario Campus Bio-Medico, 00128 Rome, Italy; alberto.ragusa@policlinicocampus.it (A.R.); francesco.tedesco@policlinicocampus.it (F.T.); f.prata@policlinicocampus.it (F.P.); rocco.papalia@policlinicocampus.it (R.P.); 4 Department of Neuroscience, Reproductive Sciences and Dentistry, University of Naples “Federico II”, 80138 Naples, Italy; felice.crocetto@unina.it (F.C.); ciro.imbimbo@unina.it (C.I.)

**Keywords:** retroperitoneal, Hugo RAS, RAPN, pyeloplasty

## Abstract

**Objectives:** To describe the surgical setup, docking strategy, and perioperative outcomes of retroperitoneal renal surgery performed with the Hugo™ RAS system, reporting, to our knowledge, the first dedicated surgical series. **Methods:** Between February and August 2025, 30 consecutive patients underwent retroperitoneal renal surgery with the Hugo™ RAS system at a single institution. Procedures included robot-assisted partial nephrectomy (RAPN, n = 15) and robot-assisted pyeloplasty (n = 15), performed by a single experienced surgeon. A standardized retroperitoneal access and reproducible trocar configuration were adopted. A three-arm configuration with predefined docking and tilt angles was used. Feasibility was defined as completion of the procedure without conversion to open or transperitoneal surgery. Perioperative data were prospectively collected and reported as median and interquartile range (IQR). **Results:** All procedures were completed without conversion or additional trocar placement. In the RAPN cohort, median docking time was 5 min (IQR 5–5), operative time was 180 min (IQR 172–200), and estimated blood loss was 200 mL (IQR 150–250). Three patients (20%) experienced Clavien–Dindo grade II complications; no major complications occurred. Positive surgical margins were observed in 1 patient (6%), with no radiological recurrence at 6-month follow-up. In the pyeloplasty cohort, operative time was 150 min (IQR 147–180) and estimated blood loss was 70 mL (IQR 50–100). One patient (7%) developed a grade II complication. Renal function remained stable in both groups. **Conclusions:** Retroperitoneal renal surgery using the Hugo™ RAS system is feasible, safe, and reproducible when supported by a standardized docking and port configuration.

## 1. Introduction

Minimally invasive renal surgery has undergone substantial evolution over the past two decades, with robotic platforms progressively expanding the indications and technical standardization of a broad range of renal procedures [1]. Although the transperitoneal route remains the most commonly adopted approach, retroperitoneal access represents a well-established alternative with distinct anatomical and perioperative advantages [2].

The retroperitoneal approach provides direct access to the kidney and renal hilum without the need for bowel mobilization, thereby limiting intraperitoneal manipulation. This characteristic may translate into reduced postoperative ileus, lower risk of intra-abdominal adhesions, and faster recovery. Furthermore, the direct posterior access is particularly advantageous for posterior renal masses and for procedures involving the renal pelvis or proximal ureter, making this approach suitable for both tumor excision and reconstructive interventions [3].

Over the years, the retroperitoneal approach has gained increasing acceptance for robot-assisted partial nephrectomy (RAPN) and pyeloplasty. In selected patients, it may facilitate direct access to the target anatomy, reduce operative dissection, and avoid the challenges associated with entering the peritoneal cavity, particularly in patients with previous abdominal surgery. Despite its potential advantages, the retroperitoneal approach remains less widely adopted than the transperitoneal route because of its technical complexity. The restricted working space, limited anatomical landmarks, and the need for precise trocar placement require meticulous preoperative planning, accurate instrument triangulation, and careful management of robotic arm positioning to avoid collisions and ensure adequate surgical exposure. In addition, the management of instrument triangulation and avoidance of arm collisions are crucial, particularly when using robotic systems based on independent arm architecture. Consequently, standardization of patient positioning, trocar placement, and docking configuration plays a central role in ensuring procedural efficiency and reproducibility [4,5,6].

The introduction of new robotic platforms, including the Hugo™ RAS system, has generated increasing interest in adapting retroperitoneal techniques to modular robotic configurations. Unlike conventional robotic systems based on a fixed patient cart, the Hugo™ RAS platform employs independent arm carts that can be positioned individually around the operating table. This modular architecture may offer greater flexibility and customization, particularly in confined anatomical spaces. However, it also requires careful planning of arm positioning, docking angles, and instrument allocation to minimize external collisions and optimize internal instrument range of motion [7].

While early clinical experiences with the Hugo™ RAS system have demonstrated its feasibility across several urological procedures, including radical prostatectomy, partial nephrectomy, and reconstructive surgery, the available literature has primarily focused on transperitoneal approaches. Consequently, evidence regarding the optimal setup and clinical performance of the platform in retroperitoneal renal surgery remains limited.

To date, dedicated clinical series specifically addressing retroperitoneal renal surgery performed with the Hugo™ RAS system are lacking. As a result, practical information regarding patient positioning, trocar configuration, docking strategy, and perioperative outcomes remains scarce.

The present study aims to describe the surgical setup, docking strategy, and perioperative outcomes of retroperitoneal renal surgery performed with the Hugo™ RAS system and, to the best of our knowledge, represents the first dedicated clinical series reported to date.

## 2. Materials and Methods

### 2.1. Study Design and Patient Population

Between February and August 2025, consecutive patients undergoing retroperitoneal renal surgery with the Hugo™ RAS system were entered into a prospectively maintained institutional database. A retrospective analysis of these patients was subsequently performed. The series included RAPN and pyeloplasties performed according to a standardized retroperitoneal technique. All procedures were carried out at Pineta Grande Hospital (Castel Volturno, Caserta, Italy) by a single surgeon with extensive experience in minimally invasive renal surgery.

Indications for surgery were established according to current European Association of Urology (EAU) guidelines and institutional protocols. Preoperative evaluation included clinical assessment and cross-sectional imaging [8,9].

Exclusion criteria included prior retroperitoneal surgery and clinically relevant spinal deformities. For RAPN, anterior renal masses were considered unsuitable for the retroperitoneal approach, while in the pyeloplasty cohort, patients with an intrarenal pelvis were excluded [10].

All patients provided written informed consent.

### 2.2. Objectives, Outcomes, and Statistical Analysis

The objective of the present study was to describe the surgical setup, trocar configuration, and docking strategy for retroperitoneal renal surgery using the Hugo™ RAS system and to evaluate its feasibility and perioperative safety.

Feasibility was defined as successful completion of the procedure without conversion to open or transperitoneal surgery. Baseline demographic, clinical, intraoperative, and postoperative data were collected. Postoperative complications were graded according to the Clavien–Dindo classification [11].

Continuous variables were reported as median and interquartile range (IQR), while categorical variables were presented as frequencies and percentages.

Statistical analyses were performed using Stata 18 (StataCorp, College Station, TX, USA).

### 2.3. Surgical Setup: Patient Positioning, Trocar Placement and Docking

After induction of general anesthesia, patients were placed in a full flank position with the umbilicus aligned at the break point of the operating table. The internal leg was flexed at approximately 45°, while the external leg was fully extended, with a pillow positioned between the legs. The operating table was flexed to maximize the distance between the costal margin and iliac crest, thereby optimizing the retroperitoneal working space.

The ipsilateral arm was positioned on an armrest secured close to the head. The patient was stabilized using two posterior supports placed at the interscapular level and at the sacral region. Additional fixation was achieved using adhesive bandages at shoulder and knee level to prevent displacement during table flexion.

A 1.5 cm oblique incision was made at the tip of the 12th rib, following the direction of the external oblique muscle fibers. Muscle fibers were bluntly separated without transection. The retroperitoneal space was initially developed with digital dissection and subsequently expanded using balloon dilation.

An 11 mm optic trocar was placed under finger guidance along the mid-axillary line over the iliac crest. An 8 mm robotic trocar was positioned along the psoas muscle below the 12th rib–vertebral angle. A 12 mm assistant trocar was placed posteriorly along the psoas muscle behind the iliac crest. Then the 12 mm Hasson trocar was finally positioned at the site of the initial incision, and the working space was created by CO^2^ inflation. A second 8 mm robotic trocar was positioned along the anterior axillary line on the same horizontal axis as the umbilicus, following laparoscopic dissection of the anterior peritoneal reflection.

**Figure 1:**
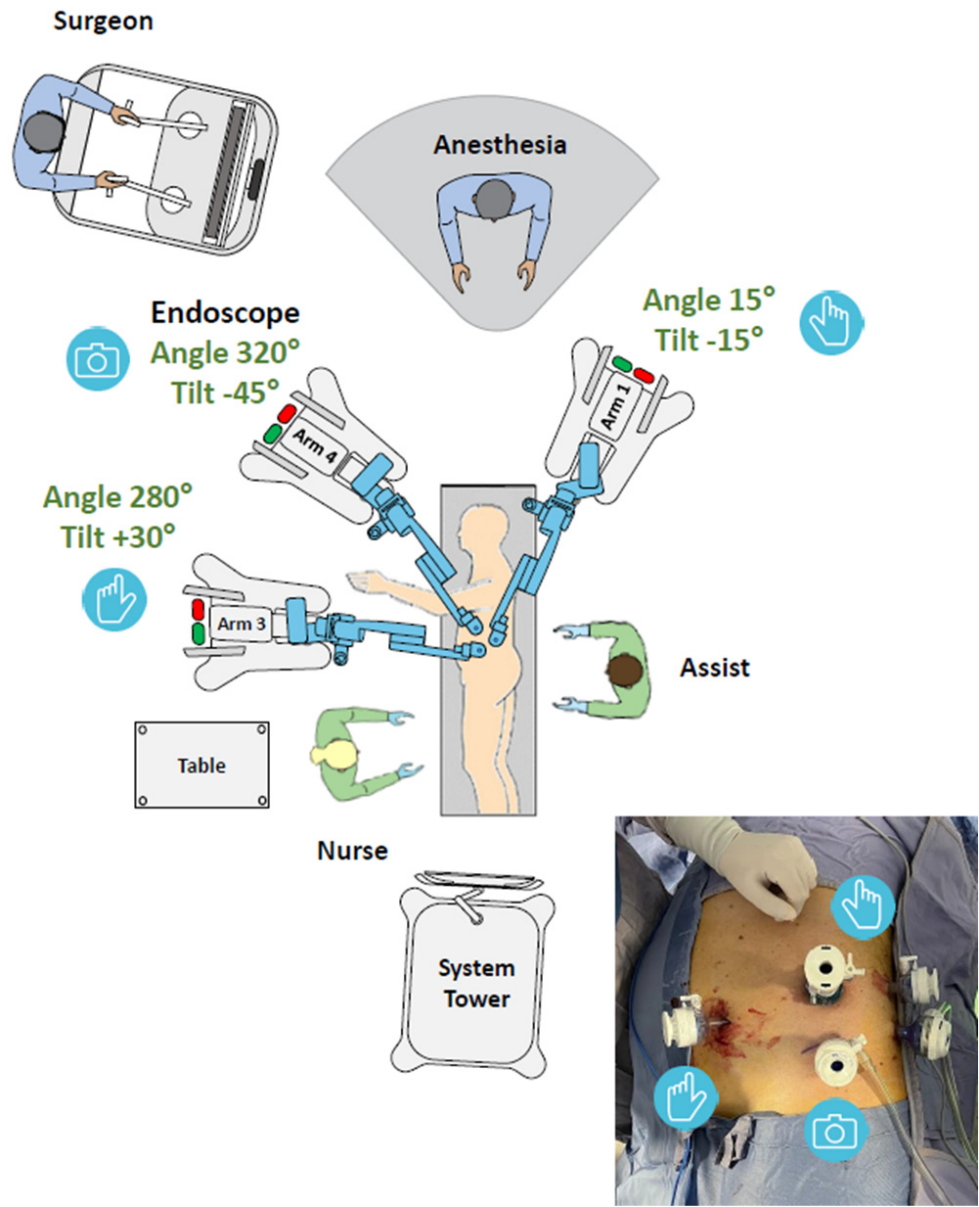
Docking angles, tilt settings, and trocar configuration for the left side.

**Figure 2:**
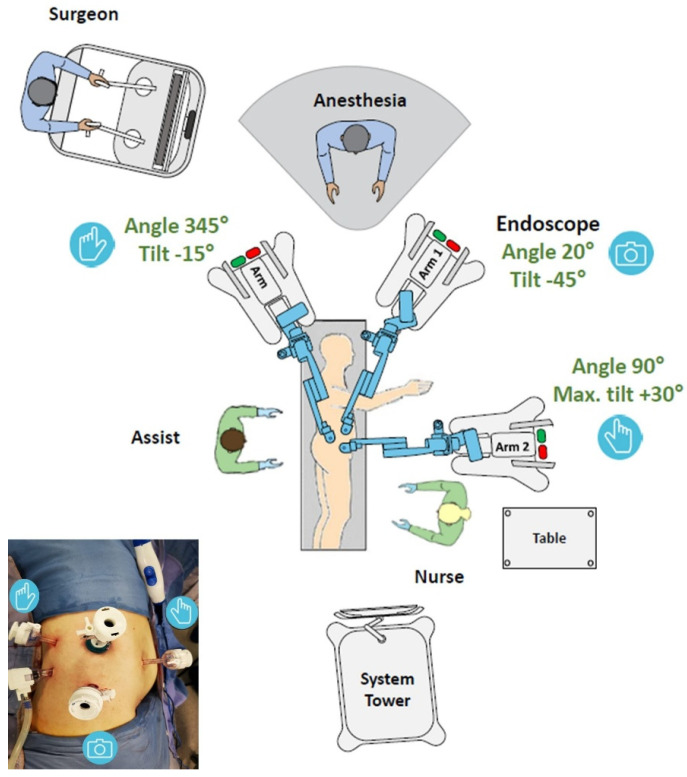
Docking angles, tilt settings, and trocar configuration for the right side.

A three-arm configuration was adopted to maximize working space for the bedside assistant and to minimize the risk of internal and external arm collisions. Based on this setup, the docking and tilt angles were reported in Figure 1 and Figure 2.

### 2.4. Surgical Technique

#### 2.4.1. Partial Nephrectomy

The right robotic arm was primarily fitted with monopolar curved scissors, which were replaced with a large needle driver during suturing. The left arm was equipped with either Cadiere or fenestrated bipolar forceps according to surgical preference. A 30° endoscope was consistently utilized throughout all procedures to optimize visualization within the retroperitoneal space.

The dissection began with mobilization of the paranephric fat, which is subsequently removed using laparoscopic forceps to further expand the operative field. The kidney, together with the surrounding adipose tissue, is first released posteriorly along the psoas muscle. Dissection then proceeded to free the upper and lower poles from their attachments to adjacent structures and, when necessary, extends anteriorly.

Gerota’s fascia was then incised to expose the perinephric fat. The renal mass was carefully identified and exposed, while the peritumoral fat is intentionally left in place.

Tumor excision was carried out with the off-clamp technique along a well-defined dissection plane. Bleeding from the tumor bed was controlled using coordinated suction and irrigation, with targeted monopolar coagulation applied for pinpoint hemostasis.

Renorrhaphy was systematically performed using a single running 2/0 Monocryl suture secured with Hem-o-lok clips according to the sliding-clip technique. After restoration of normotensive conditions and confirmation of hemostasis, adjunctive hemostatic agents (TABOTAMP™, or Floseal^®^) were applied over the resection surface as needed. A single drain was inserted under direct vision through the inferior 8 mm port, and Gerota’s fascia was subsequently closed.

#### 2.4.2. Pyeloplasty

The ureteropelvic junction was identified and dissected. A standard dismembered Anderson–Hynes pyeloplasty was performed, including excision of the stenotic segment, ureteral spatulation, and re-anastomosis to the renal pelvis using a running absorbable suture. In the presence of a crossing vessel causing extrinsic compression, the ureteropelvic junction was reconstructed with anterior (ventral) transposition to the vessel to prevent recurrent compression, while preserving vascular integrity.

A double-J ureteral stent was placed in all patients. A single drain was placed under direct visualization through the inferior 8 mm trocar site.

### 2.5. Postoperative Management and Follow-Up

Early postoperative mobilization was encouraged in all patients. The urinary catheter was removed on the first postoperative day. The abdominal drain was subsequently removed once output remained minimal for at least 12 h following catheter removal, after which patients were considered eligible for discharge.

After RAPN, a structured follow-up protocol was implemented. Laboratory evaluation, including complete blood count, serum electrolytes, and renal function tests, was performed one month postoperatively and subsequently every three months. Contrast-enhanced abdominal CT imaging was scheduled at three months after surgery and every six months thereafter.

In patients undergoing pyeloplasty, follow-up included renal ultrasonography and serum creatinine assessment at one month and 6 months after surgery. The ureteral stent was removed 45 days after surgery.

## 3. Results

A total of 30 patients were included, 15 undergoing RAPN and 15 undergoing pyeloplasty. All procedures were completed without conversion to open surgery or to a transperitoneal approach, and no additional trocars were required. No intraoperative conflicts between the robotic arms or between the robotic system and the laparoscopic assistant were observed.

### 3.1. RAPN Cohort

In the RAPN group, median age was 60 years (IQR 55–62) and the median BMI was 29 kg/m^2^ (IQR 28–33). The median ASA score was 2 (IQR 2–2), and the median Charlson Comorbidity Index was 2 (IQR 2–2). Preoperative hemoglobin was 14.45 g/dL (IQR 13.85–15.25), preoperative creatinine was 0.95 mg/dL (IQR 0.81–1.00), and median preoperative eGFR was 96.25 mL/min/1.73 m^2^ (IQR 83.53–105.72). Median docking time was 5 min (IQR 5–5), console time was 128 min (IQR 120–165), and operative time was 180 min (IQR 172–200). Estimated blood loss was 200 mL (IQR 150–250) (Table 1).

The median length of hospital stay was 3 days (IQR 3–5). Hemoglobin at discharge was 12.00 g/dL (IQR 10.30–12.90), creatinine was 1.02 mg/dL (IQR 0.85–1.22), and eGFR was 85.30 mL/min/1.73 m^2^ (IQR 78.38–88.55). Twelve patients (80%) experienced no postoperative complications, while 3 patients (20%) developed Clavien–Dindo grade II complications. One patient required blood transfusion due to postoperative anemia, and two patients received antibiotic therapy for postoperative fever. No major complications (Clavien–Dindo ≥ III) were observed (Table 2).

The median clinical tumor size was 31.5 mm (IQR 26–34.7). The median R.E.N.A.L. score was 5 (IQR 5–7). Clinical staging included 9 cT1a tumors (60%), 4 cT1b tumors (27%), and 2 cT2a tumors (13%). Final pathology revealed malignant tumors in 13 cases (87%) and benign lesions in 2 cases (13%). Histological subtypes included clear cell renal cell carcinoma in 10 patients (67%), papillary renal cell carcinoma in 3 patients (20%), and oncocytoma in 2 patients (13%). Positive surgical margins were observed in 1 patient (6%) (Table 3).

At a median follow-up of 6 months (IQR 6–7), creatinine was 0.95 mg/dL (IQR 0.80–1.10) and eGFR was 90.30 mL/min/1.73 m^2^ (IQR 80.50–90.55). No radiological recurrence was detected on follow-up CT imaging.

### 3.2. Pyeloplasty Cohort

In the pyeloplasty group, median age was 37 years (IQR 33–58) and the median BMI was 20.28 kg/m^2^ (IQR 18.59–24.44). The median ASA score was 2 (IQR 1–2), and the median Charlson Comorbidity Index was 1 (IQR 0–2). Preoperative hemoglobin was 14.00 g/dL (IQR 12.70–15.50), preoperative creatinine was 0.83 mg/dL (IQR 0.68–1.10), and median preoperative eGFR was 93.00 mL/min/1.73 m^2^ (IQR 74.40–95.38). Median docking time was 5 min (IQR 4–5), console time was 120 min (IQR 115–150), and operative time was 150 min (IQR 147–180). Estimated blood loss was 70 mL (IQR 50–100) (Table 1).

The median length of hospital stay was 3 days (IQR 3–5). Hemoglobin at discharge was 13.05 g/dL (IQR 12.07–13.17), creatinine was 0.80 mg/dL (IQR 0.60–1.01), and eGFR was 95.25 mL/min/1.73 m^2^ (IQR 81.00–105.40). Fourteen patients (93%) experienced no postoperative complications, while 1 patient (7%) developed a Clavien–Dindo grade II complication requiring antibiotic therapy for postoperative fever. No major complications (Clavien–Dindo ≥ III) were observed. At a median follow-up of 6 months (IQR 6–6), creatinine was 0.80 mg/dL (IQR 0.70–0.95) and eGFR was 96.30 mL/min/1.73 m^2^ (IQR 80.00–102.40) (Table 2). Ureteral stents were successfully removed in all patients 45 days after surgery. No patient required secondary intervention during follow-up, and postoperative ultrasonography demonstrated stable or improved hydronephrosis in all cases.

## 4. Discussion

The retroperitoneal approach has progressively redefined its role in robotic renal surgery, moving from a niche alternative to a well-established option in selected patients. Contemporary evidence suggests that, when performed within a standardized workflow, retroperitoneal access achieves surgical quality comparable to the transperitoneal route while offering potential perioperative advantages [10,12].

In a recent large meta-analysis including over 5000 patients, Shrivastava et al. demonstrated that retroperitoneal RAPN was associated with significantly shorter operative time (*p* < 0.0001), reduced overall complications (*p* = 0.01), and shorter length of hospital stay (*p* < 0.00001), without differences in major complications (*p* = 0.37) or positive surgical margins (*p* = 0.69) compared with the transperitoneal approach [13]. Similar findings were reported in a pooled analysis by Xia et al., which confirmed lower estimated blood loss (*p* < 0.00001) and shorter operative time (*p* = 0.02) for the retroperitoneal route, with no significant difference in oncological outcomes (PSM *p* = 0.13) [14]. These data collectively support the oncological safety of the retroperitoneal approach while highlighting its perioperative efficiency.

With regard to pyeloplasty, recent comparative analyses have also demonstrated equivalent functional outcomes between transperitoneal and retroperitoneal techniques. A contemporary meta-analysis reported no significant difference in success rate (*p* = 0.09) or overall complications (*p* = 0.69), although operative time differed significantly (*p* < 0.001), reflecting the technical nuances of each approach. These findings indicate that the retroperitoneal route is not inferior in terms of efficacy, but demands precise surgical planning and ergonomic optimization [15].

Importantly, most of the current evidence has been generated using the da Vinci robotic platform, where reproducibility has been achieved through highly standardized port placement and docking strategies [16,17,18]. The transition to modular robotic systems with independent arm architecture, such as the Hugo™ RAS system, introduces additional technical variables, particularly in confined spaces like the retroperitoneum. Independent arm carts require meticulous attention to spacing, angulation, and bedside ergonomics to prevent both internal and external instrument collisions [19,20,21,22,23].

Early feasibility data for Hugo™ in renal surgery have been encouraging. Prata et al., in the first reported off-clamp RAPN series performed with Hugo™ (n = 7), described a median docking time of 6 min (IQR 4–8), median console time of 83 min (IQR 68–115), no intraoperative complications, and no need for additional trocar placement, confirming technical feasibility [24]. However, structured retroperitoneal series with this platform have been lacking.

In the present study, all 30 procedures were completed without conversion to open surgery or to a transperitoneal approach, and without the need for additional port placement, supporting technical feasibility within a reproducible configuration. In the RAPN cohort, docking time was 5 min (IQR 5–5), console time was 128 min (IQR 120–165), operative time was 180 min (IQR 172–200), and estimated blood loss was 200 mL (IQR 150–250). Postoperative complications occurred in 3 patients (20%), all Clavien–Dindo grade II (one transfusion for anemia and two cases requiring antibiotic therapy for fever), with no major complications (Clavien–Dindo ≥ III). The median length of stay was 3 days (IQR 3–5), renal function remained stable at 6 months (creatinine 0.95 mg/dL, IQR 0.80–1.10; eGFR 90.30 mL/min/1.73 m^2^, IQR 80.50–90.55), and no radiological recurrence was detected on follow-up CT imaging. Positive surgical margins were observed in 1 patient (6%), consistent with contemporary robotic RAPN benchmarks.

In the pyeloplasty cohort, docking time was 5 min (IQR 4–5), operative time was 150 min (IQR 147–180), and estimated blood loss was 70 mL (IQR 50–100). One patient (7%) experienced a Clavien–Dindo grade II complication requiring antibiotic therapy for postoperative fever, while no major complications were recorded. Renal function parameters remained stable at follow-up.

Taken together, these findings indicate that retroperitoneal renal surgery with the Hugo™ RAS system can be performed safely and effectively when supported by a standardized surgical setup. Although our study is descriptive and not comparative, perioperative performance and complication rates appear consistent with contemporary robotic series reported in the literature.

This study has several limitations, including the single-center design, limited sample size, and relatively short follow-up, particularly for oncological endpoints. Moreover, the mixed inclusion of ablative and reconstructive procedures reflects real-world practice but limits procedure-specific statistical interpretation. Larger multicenter studies with longer follow-up are required to confirm long-term oncological control and functional durability.

Nevertheless, this first dedicated clinical series demonstrates that retroperitoneal renal surgery with the Hugo™ RAS system is feasible, safe, and reproducible within a structured operative setting, extending established retroperitoneal robotic principles to a modular independent-arm platform.

## 5. Conclusions

Retroperitoneal renal surgery performed with the Hugo™ RAS system appears to be a safe and feasible approach in selected patients. A standardized patient positioning, trocar configuration, and docking strategy allowed consistent operative performance without conversions or the need for additional ports, supporting the technical applicability of the platform within the confined retroperitoneal space.

Although further prospective studies with larger cohorts and longer follow-up are warranted to validate these findings, the present experience represents the first dedicated clinical series and provides evidence that this technique can be successfully implemented within a structured surgical setting by surgeons experienced in robotic retroperitoneal renal surgery.

## Figures and Tables

**Table 1:** Baseline and intra-operative data.

Variable	RAPN (n = 15)	Pyeloplasty (n = 15)
Age (years, median, IQR)	60 (55–62)	37 (33–58)
BMI (kg/m^2^, median, IQR)	29.23 (28.36–33.62)	20.28 (18.59–24.44)
ASA Score (median, IQR)	2 (2–2)	2 (1–2)
Charlson Comorbidity Index (median, IQR)	2 (2–2)	1 (0–2)
Preoperative Hb (g/dL, median, IQR)	14.45 (13.85–15.25)	14.00 (12.70–15.50)
Preoperative Creatinine (mg/dL, median, IQR)	0.95 (0.81–1.00)	0.83 (0.68–1.10)
Preoperative eGFR (mL/min/1.73 m^2^, median, IQR)	96.25 (83.53–105.72)	93.00 (74.40–95.38)
Docking Time (min, median, IQR)	5 (5–5)	5 (4–5)
Console Time (min, median, IQR)	128 (120–165)	120 (115–150)
Operative Time (min, median, IQR)	180 (172–200)	150 (147–180)
Estimated Blood Loss (mL, median, IQR)	200 (150–250)	70 (50–100)

**Table 2:** Post-operative data.

Variable	RAPN (n = 15)	Pyeloplasty (n = 15)
Length of stay (days, median, IQR)	3 (3–5)	3 (3–5)
Hb at discharge (g/dL, median, IQR)	12.00 (10.30–12.90)	13.05 (12.07–13.17)
Creatinine at discharge (mg/dL, median, IQR)	1.02 (0.85–1.22)	0.80 (0.60–1.01)
eGFR at discharge (mL/min/1.73 m^2^, median, IQR)	85.30 (78.38–88.55)	95.25 (81.00–105.40)
Clavien–Dindo Complications (n, %)		
0	12 (80%)	14 (93%)
1	3 (20%)	1 (7%)
Last Follow-up (months, median, IQR)	6 (6–7)	6 (6–6)
Creatinine at last follow-up (mg/dL, median, IQR)	0.95 (0.80–1.10)	0.80 (0.70–0.95)
eGFR at last follow-up (mL/min/1.73 m^2^, median, IQR)	90.30 (80.50–90.55)	96.30 (80.00–102.40)

**Table 3:** Oncological features and outcomes of RAPN.

Variable	RAPN (n = 15)
Clinical Tumor Size (mm, median, IQR)	31.5 (26–34.7)
Side (n, %)	
Right	9 (60%)
Left	6 (40%)
cT (n, %)	
T1a	9 (60%)
T1b	4 (27%)
T2a	2 (13%)
R.E.N.A.L. score (median, IQR)	5 (5–7)
Pathology (n, %)	
Benign	2 (13%)
Malignant	13 (87%)
Histology Subtype (n, %)	
Oncocytoma	2 (13%)
Clear Cell	10 (67%)
Papillary	3 (20%)
Positive Margins (n, %)	1 (6%)

## Data Availability

The original contributions presented in the study are included in the article; further inquiries can be directed to the corresponding author.

## Abbreviations

The following abbreviations are used in this manuscript:

IQR	interquartile range
BMI	body mass index
RAPN	robot-assisted partial nephrectomy
EAU	European Association of Urology
CCI	Charlson Comorbidity Index
